# *Ruminococcus bromii*-generated acetate alleviated *Clonorchis sinensis*-induced liver fibrosis in mice

**DOI:** 10.3389/fmicb.2025.1532599

**Published:** 2025-03-17

**Authors:** Chun Li, Changsheng Cheng, Liping Jiang, Xin Zhong, Guoyang Huang, Gang Mo, Deping Cao, Xiaohong Peng

**Affiliations:** ^1^Guangxi University Key Laboratory of Pathogenic Biology, Guilin Medical University, Guilin, China; ^2^Department of Infectious Diseases, Guidong People’s Hospital of Guangxi Zhuang Autonomous Region, Wuzhou, China; ^3^Guangxi Key Laboratory of Molecular Medicine in Liver Injury and Repair, The Affiliated Hospital of Guilin Medical University, Guilin, China

**Keywords:** *Clonorchis sinensis*, gut microbiome, SCFAs, liver fibrosis, HSCs

## Abstract

**Introduction:**

Infection with *Clonorchis sinensis* (*C. sinensis*) has the potential to induce liver fibrosis and significantly alter the gut microbiota. However, it remains unclear how these changes in the gut microbiota, through the gut-liver axis, influence the progression of liver fibrosis. Furthermore, it is uncertain whether targeting the gut microbiota, based on the concept of the gut-liver axis, could be a potential therapeutic strategy for alleviating liver fibrosis.

**Methods:**

The gut microbiota alterations in *C. sinensis*-infected mice at multiple time points were analyzed through 16S rDNA high-throughput sequencing. *Ruminococcus bromii (R.bromii)* therapeutic effect on *C. sinensis* infected mice was evaluated. Metabolic changes following produced by *R. bromii* were analyzed using short-chain fatty acids (SCFAs) metabolomics. Additionally, *R. bromii* conditioned medium (R.b CM) or its metabolites were co-cultured with two hepatic stellate cell lines (LX2 and JS1) *in vitro* to assess their anti-fibrotic effects. Finally, RNA sequencing was employed to investigate the specific mechanism by which acetate inhibits hepatic stellate cells (HSCs) activation.

**Results:**

The abundance of *R. bromii* increased during the inflammatory stage of *C. sinensis* infection and decreased significantly during the fibrosis stage. Oral gavage of *R. bromii* significantly inhibited *C. sinensis*-induced liver fibrosis while restoring the intestinal barrier. The activation of HSCs was significantly inhibited *in vitro* upon incubation with *R.b* CM. Acetate was identified as a key metabolite generated from *R. bromii* in *R.b* CM, and acetate attenuated *C. sinensis*-induced liver fibrosis *in vitro* and in vivo. Mechanistically, acetate inhibited the activation of HSCs by activating the PI3K/AKT signaling pathway to prevent the progression of liver fibrosis in mice infected with *C. sinensis*.

**Discussion:**

*R. bromii* exerted a protective effect on hepatic fibrosis by delivering acetate via the gut-liver axis to active the PI3K/AKT signaling pathway in HSCs. Furthermore, *R. bromii* can be used as a probiotic therapy to alleviate hepatic fibrosis.

## 1 Introduction

Clonorchiasis is a parasitic disease caused by the infection of *Clonorchis sinensis* (*C. sinensis*), which is mainly caused by the consumption of raw or undercooked freshwater fish and other aquatic animals. This condition is prevalence across East Asia, particularly in China, South Korea and Vietnam, with an estimated global prevalence of 35 million individuals, among whom 15 million reside in China (Na et al., 2020). Clonorchiasis primarily targets the liver, causing significant damage, particularly in the form of fibrosis within the hepatobiliary duct. If left untreated, this condition can progress to the more severe stage of hepatobiliary cancer. Consequently, *C. sinensis* has been classified as a type I biological carcinogen, directly linked to the development of causing cholangiocarcinoma (Bouvard et al., 2009; Choi et al., 2004). In recent years, liver fibrosis has garnered increasing attention due to its potential reversibility (Lee et al., 2015). However, despite this promising aspect, there remains a pressing need for innovative safe therapeutic strategies to effectively treat and reverse liver fibrosis, as no such drug currently exists.

The primary driving force behind liver fibrosis caused by *C. sinensis* stems from its excretory-secretory products (Cs ESPs), which act as potent stimulators of HSCs activation and subsequent differentiation into myofibroblasts, unleashing a cascade of fibrosis responses (Hu et al., 2009). Chronic infestation with *C. sinensis* gives rise to biliary duct obstruction, stemming from the relentless mechanical irritation by the parasite’s body, disrupting bile acid flow and precipitating bile stasis, thereby impeding the enterohepatic circulation (Lun et al., 2005). Moreover, during its parasitic tenure, the worm continuously deposits eggs and releases ESPs into the intestinal milieu, profoundly altering the gut microbiota landscape of the host and disrupting the delicate equilibrium within the intestinal microbial community. Emerging research underscores the potential implications of post-liver fluke infection gut microbiome alterations on liver fibrosis. For instance, *C. sinensis*-infected individuals exhibit heightened bacterial diversity coupled with a depletion of *Bacteroides* and *Bifidobacterium* species, and was negatively correlated with *C. sinensis* eggs per gram (EPG). While *Dorea*, a pro-inflammatory bacterium, emerged as the dominant taxon (Xu et al., 2018). In a hamster model of *Opisthorchis viverrini* (*O. viverrini*) infection, another liver fluke, metabolomics combined with fecal microbiota was used to analyze the metabolism and gut microbial spectrum of hamsters at different stages of infection (Haonon et al., 2021). The results showed that in the acute infection phase (1 month post infection), the liver inflammation and injury, increased intestinal microbial diversity, *Akkermansia* abundance and serum 4-aminobutyric acid accumulation were observed. And in the chronic phase (4 months post infection) hepatic peribiliary fibrosis was observed. The decrease of intestinal diversity and *Akkermansia* abundance was consistent with that in the uninfected mice. While 4-aminobutyric acid had been reported to have a protective effect on liver injury (Hata et al., 2019), and *Akkermansia mucinipgila* had been shown to reduce CCL4-induced liver fibrosis, enhanced intestinal barrier and regulated inflammatory response in mice (Keshavarz Azizi Raftar et al., 2021). It is speculated that parasite infection triggers a compensatory process in the host organism to protect the liver. Therefore, the gut microbiota at different stages of parasite infection deserves further study. Collectively, parasitic infections introduce profound perturbations to the gut microbiota of their hosts, fostering ecological imbalances. However, it is still unclear whether there are differences in the intestinal flora of hosts during the early stage of *C. sinensis* infection and the onset of fibrosis, and if so, whether these differences are related to its pathogenicity.

In this study, we investigated the changes in the gut microbiota during the acute and fibrotic stages of *C. sinensis* infection. We identified a specific bacterium, *Ruminococcus bromii* (*R. bromii*), that was involved in the progression of *C. sinensis* infection, and elucidated how *R. bromii* affected the development of liver fibrosis and intestinal barrier damage caused by *C. sinensis*. This study provides potential therapeutic target for the treatment of *C. sinensis*-related liver fibrosis.

## 2 Methods

### 2.1 *Clonorchis sinensis* infection and high-throughput sequencing of 16S rDNA

Fish infected with *C. sinensis* were purchased from Heng County, Guangxi, China. These fish were then digested with pepsin, centrifuged and resuspended in 100 μL PBS with 150 metacercariae for subsequent gavage.

*BALB/c* mice were selected and randomly divided into three groups, consisting of six mice per group. Each mouse in the infected group was intragastrically administered with 150 *C. sinensis* metacercariae. The colonic contents of the mice were collected at the 4th week and 16th week post-infection, respectively. Meanwhile, the colonic contents of normal control mice were collected for comparative analysis. The Cetyltrimethylammonium Bromide (CTAB) method was used to extract DNA from the colonic contents, and the V3-V4 region of 16S rRNA was amplified. Sequencing was performed using the NovaSeq 6000 platform. Post-sequencing data were filtered and denoised using QIIME 2 to obtain ASV (Amplicon Sequence Variant) feature sequences. Species annotation was conducted using the SILVA database with NT-16S. Subsequent analyses, including α-diversity, β-diversity, species differentiation, and Lefse analysis, were performed using R software (version 4.3.0).

### 2.2 Animal model constructed

Female *BALB/c* mice aged 6–8 weeks were randomly divided into three groups. Mice in the infection group were administered 100 μL of metacercariae suspension via gavage. Mice in the *R.b* group were subjected to a daily gavage of *R. bromii* (1 × 10^9^ colony forming units (CFUs)/200 μL/per mice). Normal Chow (NC) and *C. sinensis* group were given an equal volume of PBS solution by gavage every day.

For *C. sinensis*-infected mice treated with acetate, 200 mM sodium acetate was added to their daily drinking water post-infection (Qi et al., 2023). Mice in the NC group and the *C. sinensis*-only group were provided with double-distilled water as the drinking solution. After 8 weeks of feeding, the mice were sacrificed, and serum, tissue and fecal samples were collected for further analysis. All animal experiments were conducted in accordance with the guidelines for the Care and Use of Laboratory Animals in China, and approved by the ethical committee for animal research of Guilin Medical University (approval No. GLMC20230732).

### 2.3 Histology

The right lobe of the liver was fixed in 4% PFA for 48 h, followed by dehydration and embedding in paraffin. Sections were cut to 4 μm thick, and the liver was stained with hematoxylin-eosin (HE), Masson, and Sirius red trichrome, and the colon tissue was stained with HE and Alcian Blue Periodic Acid-Schiff (AB-PAS) stain. Images were captured using an Olympus BX53 microscope. Quantitative analysis of Masson trichrome staining and Sirius red staining was performed using ImageJ software.

### 2.4 Biochemical assays

The serum levels of alanine aminotransferase (ALT), aspartate aminotransferase (AST), and hydroxyproline (HYP) were assessed using the ALT kit (cat no. C009-2-1, Jiancheng, China), AST kit (cat no. C010-2-1, Jiancheng, China), and HYP kit (cat no. A030-2-1, Jiancheng, China) according to the manufacturer’s instructions. Acetate mouse ELISA kit was used to detect acetate levels in the serum of mice.

### 2.5 Bacterial culture

*Ruminococcus bromii* ATCC51896 was anaerobically cultured in chopped meat carbohydrate medium at 37°C for 48 h. Once the OD600 of the culture reached 1, the culture broth was centrifuged at 5,000 g for 10 min at 4°C. Subsequently, the supernatant was filtered through a 0.22 μm filter to obtain the *Ruminococcus bromii* conditioned medium, henceforth referred to as *R.b* CM. The cells collected by centrifugation were washed three times with sterile anaerobic PBS and resuspended for oral administration in mice.

### 2.6 Cell culture and treatment

The human hepatic stellate cell line LX2 and the mouse hepatic stellate cell line JS1 were cultured in DMEM supplemented with 10% FBS and 1% penicillin/streptomycin at 37°C and 5% CO_2_ until they reached 70–80% confluence. Subsequently, the medium was exchanged with serum-free medium to induce cellular starvation overnight. Afterwards, the control group was replenished with DMEM containing 10% FBS, while the experimental groups were incubated in DMEM supplemented with 50 μg/mL of ESPs and *R.b* CM, or sodium acetate (Sigma S2889) for 48 h. ESPs were extracted as described previously (Jin et al., 2014).

### 2.7 Cell proliferation assay (CCK8)

LX2 and JS1 cells were seeded in a 96-well plate at densities of 2 × 10^3^/100 μL and 3 × 10^3^/100 μL, respectively. Once the cells adhered, they were treated with *R.b* CM at different concentrations (10, 5, 2.5, and 1.25%) for 48 h. After this treatment period, the media was aspirated, and a solution composed of CCK-8 reagent and DMEM, in a 1:10 ratio, was added into each well. The plates were then incubated in a dark environment for 1 h. Finally, the optical density (OD) at 450 nm was measured for each group to calculate the cell proliferation rate.

### 2.8 Immunofluorescence staining

Prepared cell slides and fixed the treated cells in 4% PFA. Permeabilized the cells with 0.5% Triton X-100 for 20 min and then blocked with 5% BSA at room temperature. Incubated overnight with α-SMA antibody (Abclonal) at a dilution of 1:200 at 4°C. Incubated with secondary antibody F488 (Proteintech) diluted in 5% BSA at a ratio of 1:500, and incubated in the dark at 37°C for 1 h. After washing with PBS, stained the nuclei with DAPI and observed the cells under a fluorescence microscope (Olympus).

### 2.9 Targeted short-chain fatty acids metabolomic analysis

Initially, pipetted 200 μL of conditioned medium or blank medium into a 1.5 mL centrifuge tube. Next, added 50 μL of 50% sulfuric acid solution and 200 μL of ether solution. Shake vigorously for 10 min, then ultrasonicated for 1 min. Centrifuged the mixture at 120,000 rpm for 20 min at 4°C. Carefully transferred the ether layer of the supernatant through anhydrous sodium sulfate before measurement. The concentration of short-chain fatty acids (SCFAs) was determined using gas chromatography–mass spectrometry (GC–MS). The raw data obtained from GC–MS was processed using ChromaTOF software (version 5.51, Leco Corp., United States) for peak integration, calibration, and quantification of each metabolite. iMAP software was used for subsequent PCA, and biomarker heatmap statistical analyses.

### 2.10 RNA extraction and real-time PCR analysis

Total RNA was extracted from mouse liver tissue using TRIzol reagent (Takara, 9109). The quality and quantity of RNA were determined using a spectrophotometer. Complementary DNA was synthesized from 1,000 ng total RNA using the Complementary DNA Transformation Kit (cat no. MR0511; Monad). Real-time PCR was performed using SYBR Green qPCR Master Mix (cat no. MQ20301S; Monad). DNA was extracted from fecal samples using a kit (Solarbio), the collected DNA was processed according to the kit instructions for qRT-PCR, ormalized against 16 s rRNA. All the gene expression levels were calculated and quantified using the 2^−ΔΔCT^ method.

### 2.11 RNA sequencing and analysis

Trizol Reagent was used to extract total RNA of JS1 cell line for RNA sequencing (Illumina NovaSeq 6000 sequencing system), and Agilent 2100 bioanalyzer was used to detect the quality of RNA. After qualified quality control, the sequencing library was constructed. Library construction and RNA sequencing were performed by Novogene (Beijing, China). Data were expressed as Fragments Per Kilobase of transcript sequence per Millions base pairs (FPKM) and using DESeq2 (version 1.20.0). log2 fold change >1, *p* value < 0.05 and KEGG enrichment was performed using clusterProfile software (version 3.8.1).

### 2.12 Western blot

Total protein was extracted using RIPA lysis buffer and quantified by the BCA method. The protein samples were then electrophoresed in an 8% or 10% SDS polyacrylamide gel and transferred to a 0.45 μm PVDF membrane. The membrane was blocked at room temperature for 2 h, followed by overnight incubation at 4°C with the primary antibody. After the primary antibody incubation, the strips were washed with TBST and incubated with a secondary antibody (1:5,000, cat no. RGAR001; Proteintech) at room temperature for 1 h. Subsequently, the strips were then developed using ECL reagent, and images were captured and analyzed for band density using Image Lab software (6.0.1). The primary antibodies employed in this study included α-SMA (cat no. A17910; Abclonal), CollagenI (cat no. 160043; Abcam), CollagenIII (cat no.184993; Abcam), Occludin (cat no. A24601; Abclonal), Claudin-1 (cat no. A21971; Abclonal), phospho-AKT (cat no. 80455-1; Proteintech) and AKT (cat no. 60203-2; Proteintech), phospho-PI3K (cat no. HA721672; Huabio) and PI3K (cat no. ET1608-70; Huabio), and GAPDH (cat no. AF7021; Affinity), all of which were diluted to a concentration of 1:1,000. GAPDH was used as the internal control.

### 2.13 Statistical analysis

Comparisons of numerical variables between the three groups were conducted using the Kruskal-Wallis test or Ordinary one-way ANOVA. The expression of total proteins was analyzed using two-way ANOVA or unpaired *t* test. All statistical analyses in this study were performed using GraphPad Prism 9.0 software or R software (version 4.3.0). Continuous variables were presented as the mean ± standard deviation (SD). When *p* < 0.05, the difference was considered statistically significant.

## 3 Results

### 3.1 The abundance of *Ruminococcus bromii* undergo a notable decline during the fibrotic phase of *Clonorchis sinensis* infection

**Figure 1:**
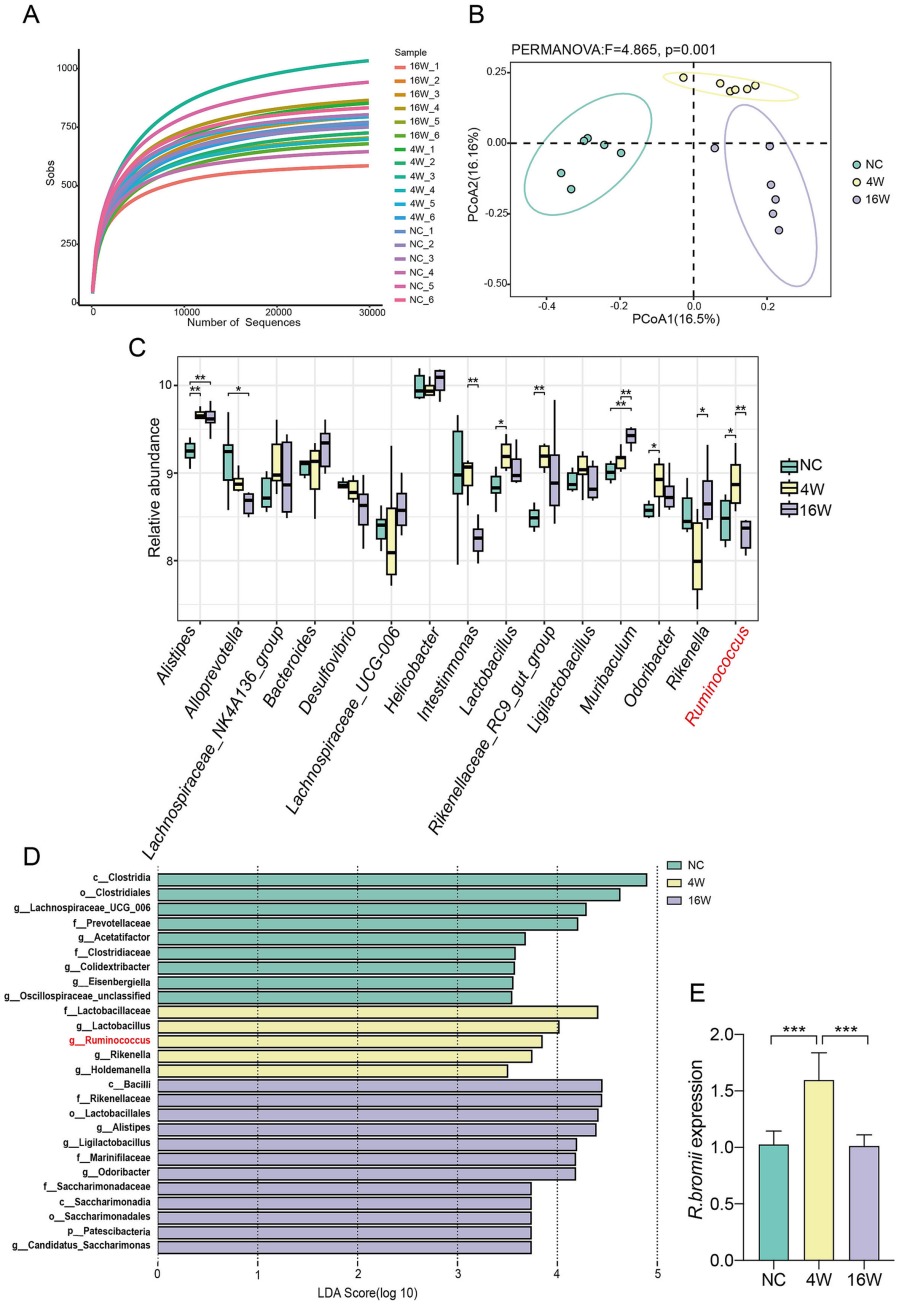
Dynamic changes of *R. bromii* abundance during *C. sinensis* infection. **(A)** Rarefaction curves representing the gut microbiota diversity of mice from the NC, 4W and 16W groups (*n* = 6 per group). **(B)** A PCoA plot constructed using the Binary Jaccard algorithm, with distinctively colored dots representing the different groups. **(C)** The relative abundance of the top 15 genera associated with infection. **(D)** Distribution histograms showing the gut bacterial species enriched during the infection process, with an LDA value > 3.5. **(E)** qPCR validation of the relative abundance of *R. bromii* in the feces of the three groups of mice. Significant differences were analyzed using the Kruskal Wallis test **(C)** and Ordinary one-way ANOVA **(E)**. *p < 0.05, **p < 0.01, ***p < 0.001.

To delve into the intricacies of gut microbiota in mice infected with *C. sinensis*, we performed high-throughput sequencing of 16S rDNA derived from their colonic contents. The saturation of rarefaction curves for these samples underscored the adequacy of sequencing depth, ensuring the detection of a robust bacteria repertoire (Figure 1A). By leveraging Principal Coordinate Analysis (PCoA) to assess β-diversity, we quantitatively evaluated the similarity and dissimilarity among microbial communities, unveiling pronounced variations in microbial composition among the three experimental groups (Figure 1B). Our subsequent screening of the top 15 genera associated with infection, revealed statistically significant changes in nine of them. Notably, as the infection progressed, conditionally pathogenic bacteria such as *Alistipes* and *Muribaculum* exhibited an upsurge in their abundance. However, an increase in *Lactobacillus* and *Odoribacter* was exclusive to the gut microbiota infected for 4 weeks (Figure 1C). Remarkably, *Ruminococcus* displayed a distinctive temporal pattern, peaking in abundance at 4 weeks post infection before tapering off at 16 weeks. This pattern was reinforced by Lefse analysis, highlighting the unique enrichment of *Ruminococcus* solely at the 4-weeks mark (Figure 1D). Among the *Ruminococcus* genus, *Ruminococcus bromii* (*R. bromii*), a pivotal species, emerged as a subject of particular interest for further investigation. To validate the fluctuations in *R. bromii* abundance, we employed qRT-PCR to detect its expression in fecal samples of the three groups. The results concurred with our initial findings, confirming that *R. bromii* abundance indeed increased at 4 weeks and significantly decreased at 16 weeks (Figure 1E). Integrating these insights, we speculated that *R. bromii* may play a pivotal, potentially protective role during the fibrotic phase of *C. sinensis* infection.

### 3.2 Oral administration of *Ruminococcus bromii* alleviates *Clonorchis sinensis* induced liver fibrosis in mice

**Figure 2:**
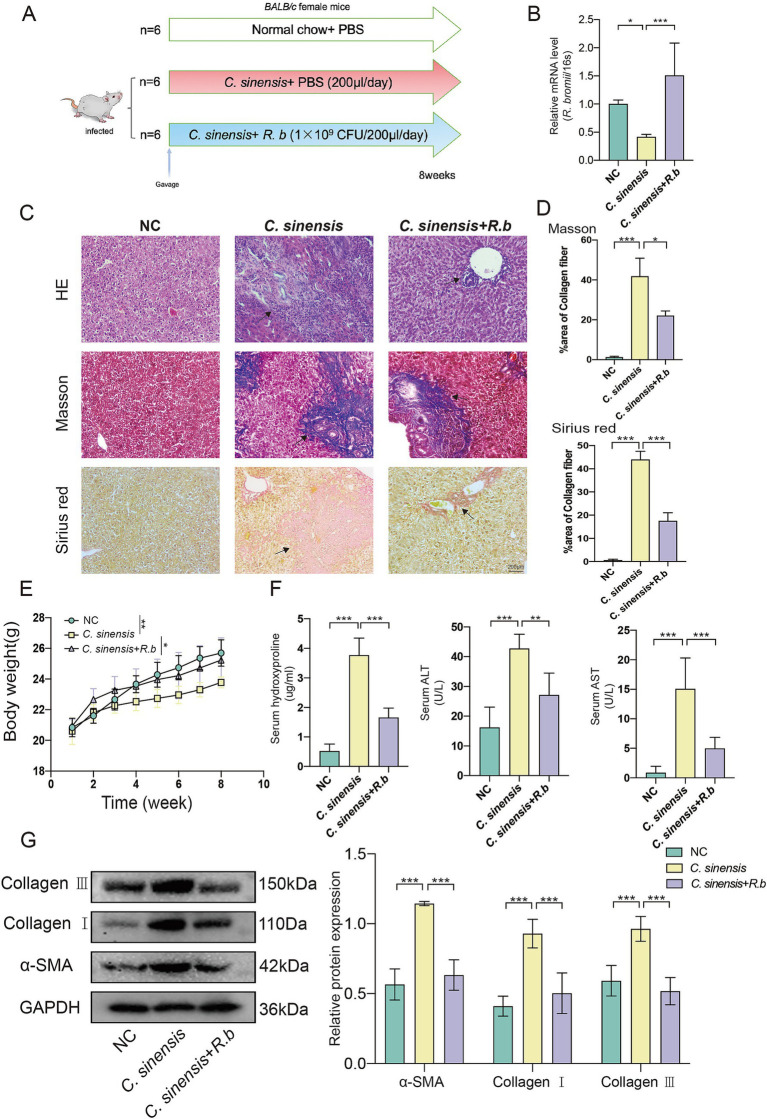
*R. bromii* protects against liver fibrosis formation in the mouse model of *C. sinensis* infection. **(A)** Experimental design for the *R. bromii* gavage in mouse model. **(B)** Gene expression of *R. bromii* in the feces of the three groups of mice. **(C,D)** Staining of liver pathological sections from three groups of mice (HE, Masson, and Sirius red, scale bar 200 μm), black arrows indicate collagen fiber deposition. And data for Masson and Sirius red staining are presented as the mean ± SD of three independent experiments. **(E)** The body weight gain of three groups. **(F)** HYP, ALT, and AST levels in the serum of mice. **(G)** The expression levels of α-SMA, Collagen I and Collagen III proteins. Significant differences were analyzed using Ordinary one-way ANOVA **(B,D,F)** and two-way ANOVA **(E,G)**. *p < 0.05, **p < 0.01, ***p < 0.001.

To explore the effect of *R. bromii* supplementation on *C. sinensis* infection in mice, we established a *BALB/c* mouse model by gavage of 150 *C. sinensis* metacercariae per mouse, followed by daily gavage of either *R. bromii* (1 × 10^9^ CFU/mL) or PBS post infection (Figure 2A). Our results confirmed the successful colonization of *R. bromii* in mice for 8 weeks, as evidenced by qPCR analysis (Figure 2B). Notably, we observed a marked decline in the body weight of infected mice compared to the NC group, whereas *R. bromii* intervention slightly mitigated this weight loss (Figure 2E). Furthermore, serum hydroxyproline (HYP) levels surged in infected mice but underwent a significantly reduction in the *R. bromii* treated group, indicative of its potential to curb collagen synthesis associated with fibrosis. Moreover, *R. bromii* also significantly reduced serum levels of aminotransferase (ALT) and aspartate aminotransferase (AST) (Figure 2F). Histopathological examination of liver sections via HE staining revealed a stark contrast between the orderly hepatocyte arrangement and normal liver structure of the NC group and the disorganized, inflammatory-ridden state of the *C. sinensis*-infected group. Encouragingly, *R. bromii* treatment visibly reduced the lesion area (Figure 2C). To assess liver collagen deposition, we employed Masson’s trichrome and Sirius red staining, which unanimously demonstrated that *R. bromii* significantly reduced the area of collagen fiber deposition (Figure 2D). Additionally, the heightened expression of fibrosis markers such as α-SMA, Collagen I and Collagen III proteins in the *C. sinensis* infection group was markedly reduced upon *R. bromii* treatment (Figure 2G). This was also consistent with the expression of these genes at the transcriptional level (Supplementary Figure S1A). These results suggested that *R. bromii* has an inhibitory effect on *C. sinensis* induced liver fibrosis.

### 3.3 *Ruminococcus bromii* regulates intestinal inflammation and improves intestinal barrier function

**Figure 3:**
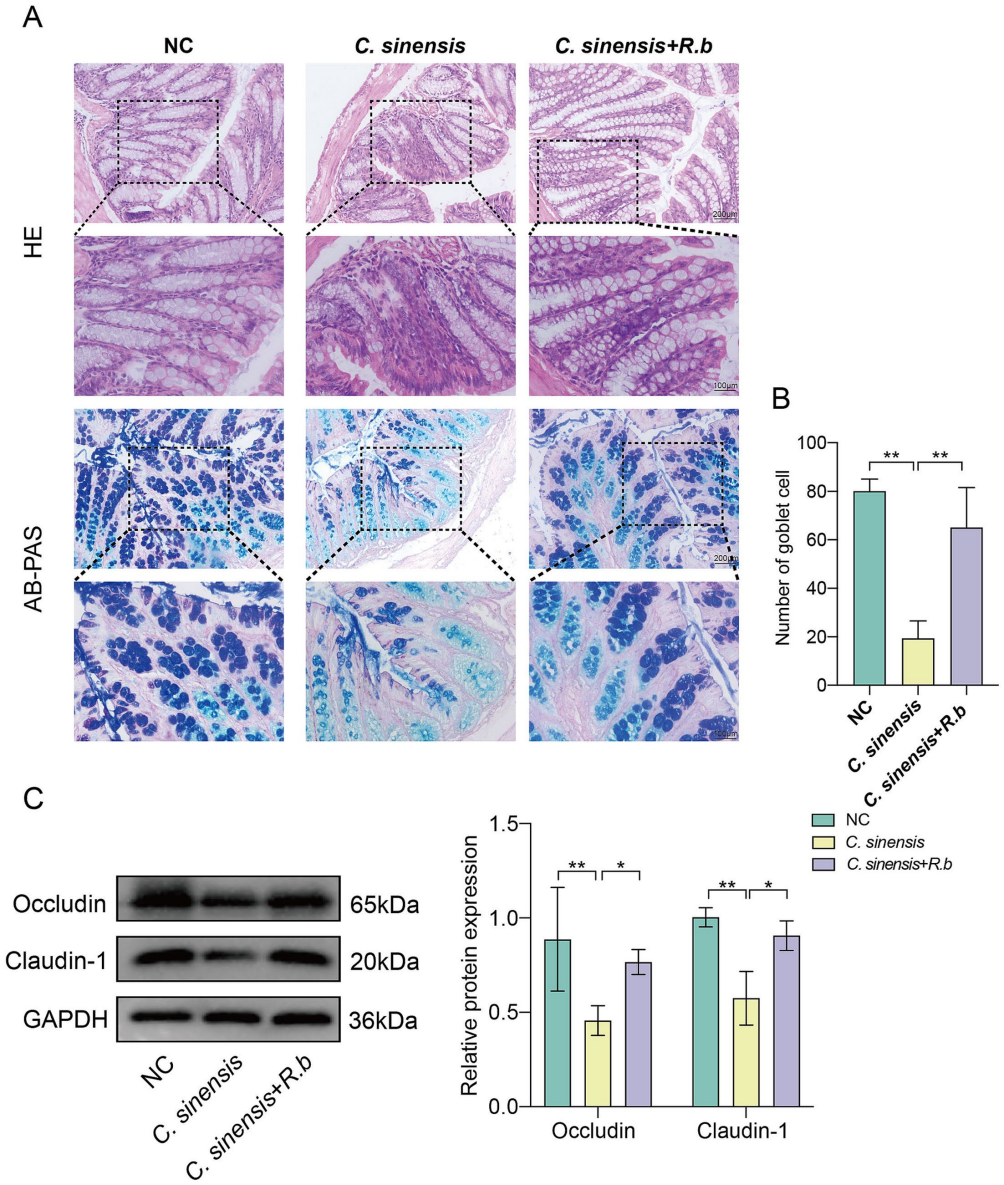
*R. bromii* improves intestinal barrier dysfunction. **(A)** Staining of HE and AB-PAS pathological sections of the mouse colon (Scale bar, 200 μm and 100 μm). **(B)** Number of goblet cells in the colon of the three groups of mice. **(C)** Expression levels of intestinal tight junction proteins Occludin and Claudin-1. Significant differences were analyzed using Ordinary one-way ANOVA **(B)** and two-way ANOVA **(C)**. *p < 0.05, **p < 0.01.

The analysis of pathological sections revealed profound alterations in the colon crypts of mice in *C. sinensis* infection group, characterized by their shallowness, the absence of goblet cells, and a conspicuous mucosal wall corrugation accompanied by extensive infiltration of inflammatory cells (Figure 3A). These findings underscored the capacity of *C. sinensis* infection to induce pathological changes in colon tissue, thereby compromising intestinal integrity. Notably, however, the deleterious effects were markedly mitigated intervention of *R. bromii*. Furthermore, employing the AB-PAS staining technique, we quantified goblet cells in the colon, revealing that *R. bromii* exhibited a remarkable ability to replenish the depleted goblet cells count (Figure 3B). Additionally, our analysis delved into the expression patterns of critical intestinal tight junction proteins, Occludin and Claudin-1. We observed that *C. sinensis* infection significantly downregulated the expression of these proteins, while *R. bromii* treatment counteracted this trend, upregulating their expression (Figure 3C). These findings underscored the potential therapeutic role of *R. bromii* in mitigating the adverse consequences of *C. sinensis* infection on intestinal architecture and function.

### 3.4 The conditioned medium of *Ruminococcus bromii* inhibits the activation of HSCs induced by Cs ESPs *in vitro*

**Figure 4:**
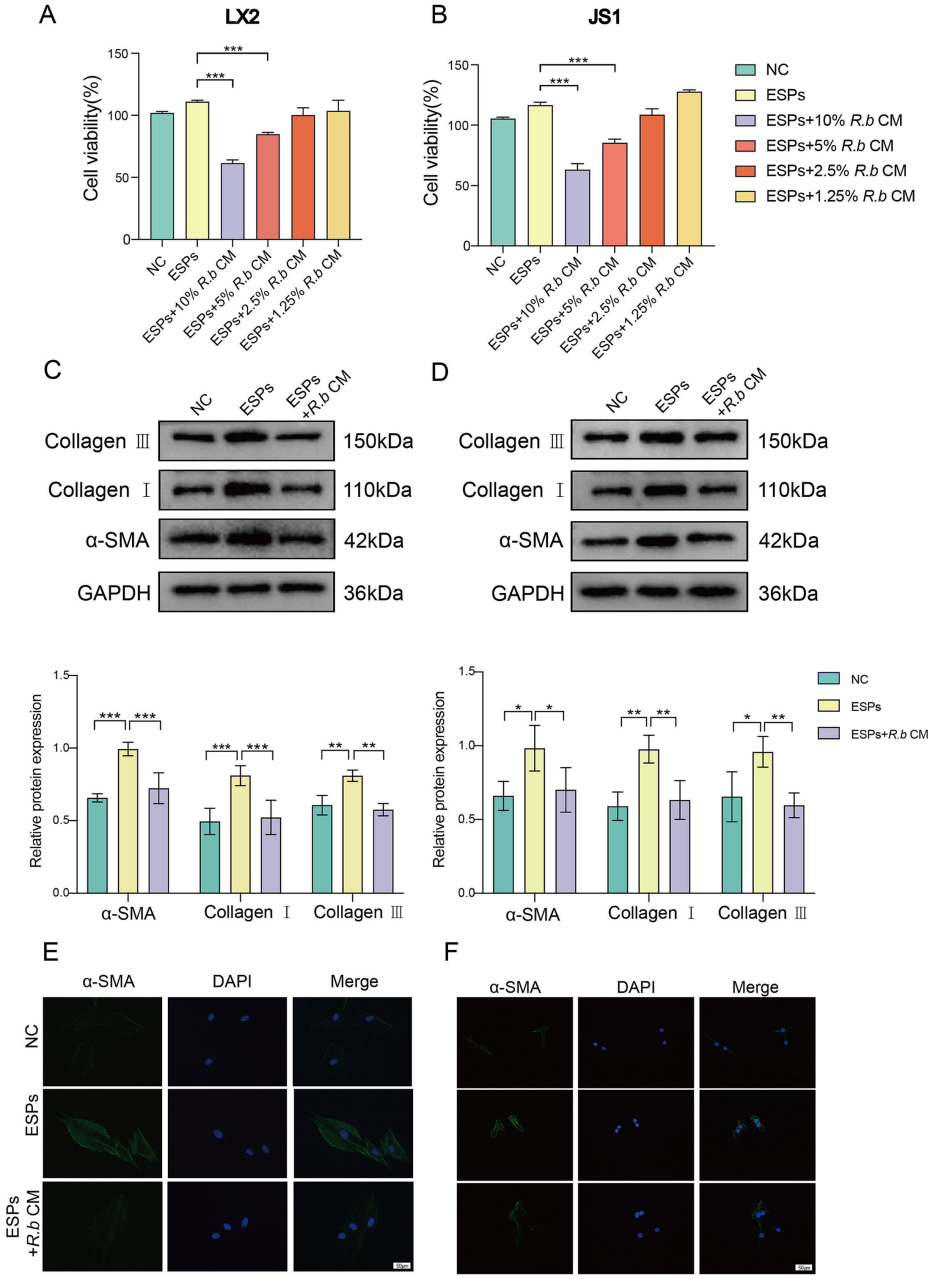
Inhibition of LX2 and JS1 cell activation by conditioned medium derived from *R. bromii*. **(A,B)** The viability of LX2 and JS1 cells was assessed after exposure to different concentrations (10, 5, 2.5, 1.25%) of *R.b* CM. **(C,D)** Following treatment with 10% *R.b* CM, the expression levels of α-SMA, Collagen I and Collagen III proteins were examined in LX2 and JS1 cells. **(E,F)** Immunofluorescence staining of α-SMA (green) in LX2 and JS1, with DAPI staining the nuclei blue. Significant differences were analyzed using Ordinary one-way ANOVA **(A,B)** and two-way ANOVA **(C,D)**. *p < 0.05, **p < 0.01, ***p < 0.001.

We initially used ESPs to activate HSCs in vitro, including both the human LX2 cell line and mouse JS1 cell line. Subsequently, we exposed these cells to varying concentrations of *R. bromii* conditioned medium (*R.b* CM). The ensuing analysis unveiled a pronounced, concentration-dependent suppression of HSCs proliferation by *R.b* CM. Specifically, when compared to ESPs-treated cells, the survival rates of LX2 exposed to 10 and 5% *R.b* CM diminished to 61 and 84% respectively, while similar reductions were observed in JS1 cells, with survival rates declining to 63 and 85% (Figures 4A,B). Furthermore, *R.b* CM emerged as a modulator of fibrosis-related protein expression, downregulating the levels of α-SMA, Collagen I and Collagen III (Figures 4C,D). This observation was further validated through immunofluorescence assays, which demonstrated a decrease in α-SMA fluorescence intensity within HSCs treated with 10% *R.b* CM (Figures 4E,F). Collectively, these results suggested that *R.b* CM possessed the capacity to suppress the proliferation and activation of HSCs, ultimately leading to a reduction in collagen deposition.

### 3.5 The acetate produced by *Ruminococcus bromii* consistently inhibits the activation of HSCs

**Figure 5:**
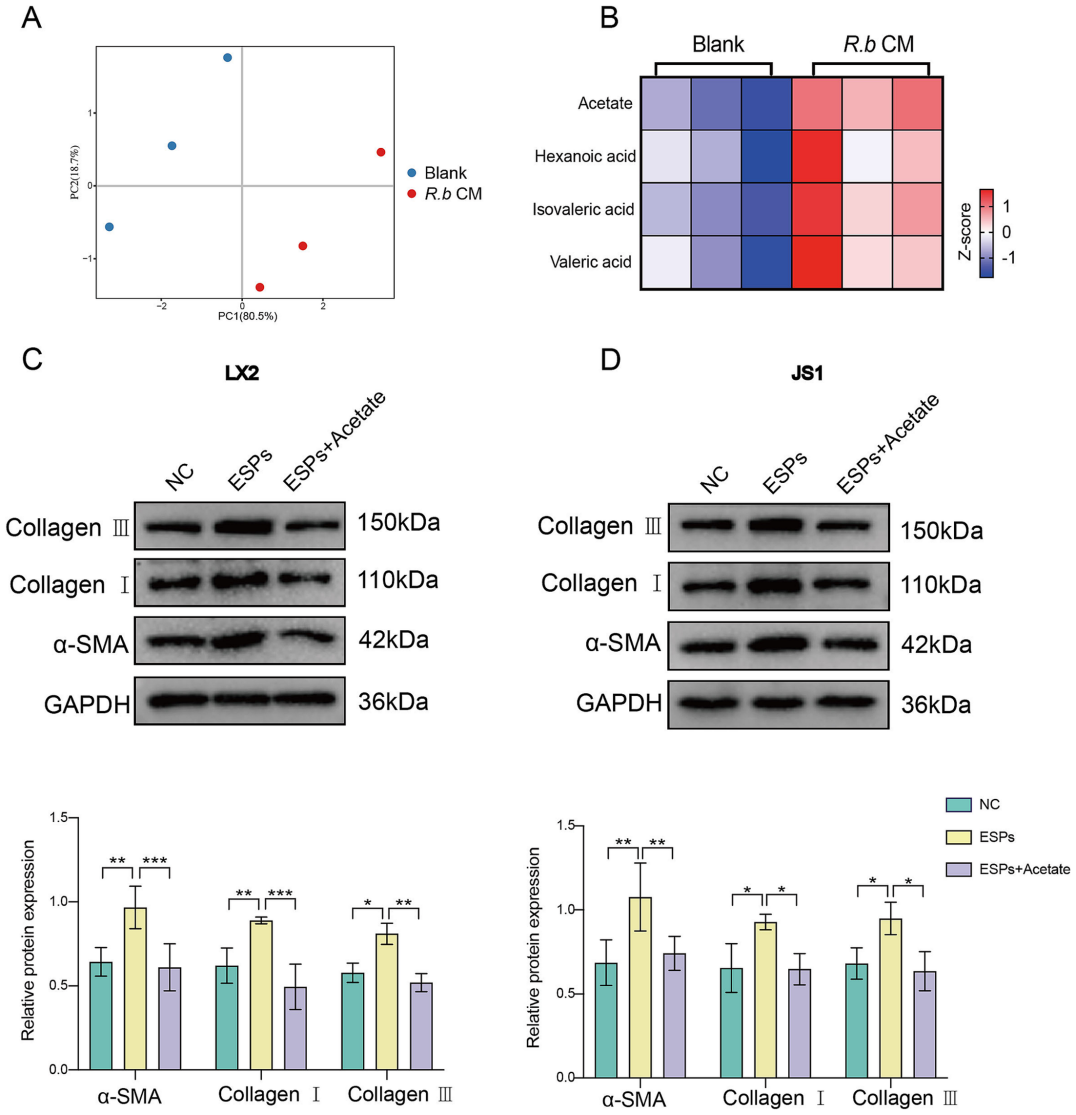
*R. bromii*-generated acetate consistently inhibits the activation of HSCs *in vitro*. **(A,B)** PCA plot and heatmap showing differences in SCFAs enrichment between the *R.b* CM group and the Blank medium group, with each group *n* = 3; **(C,D)** Expression of α-SMA, Collagen I and Collagen III proteins in LX2 and JS1 after treatment with sodium acetate at concentrations comparable to 10% *R.b* CM. Significant differences were analyzed using two-way ANOVA **(C,D)**. *p < 0.05, **p < 0.01, ***p < 0.001.

Considering that *R. bromii* is renowned for its production of short-chain fatty acids (SCFAs) (Baxter et al., 2019; Vital et al., 2018), we performed a targeted metabolic analysis focusing on SCFAs within *R.b* CM. Our analysis revealed significantly differences in the metabolic components of *R.b* CM compared to the blank medium control, with acetate standing out as the preeminent metabolite within *R.b* CM (Figures 5A,B). To validate the functional role of acetate, we treated LX2 and JS1 cells with sodium acetate, a stable form of acetate. Notably, acetate similarly reduced the expression of the fibrotic proteins α-SMA, Collagen I and Collagen III (Figures 5C,D). Overall, these findings underscored the pivotal role of acetate as a functional metabolic byproduct of *R. bromii*, effectively inhibiting the activation of HSCs induced by ESPs.

### 3.6 Acetate attenuates liver fibrosis in *Clonorchis sinensis* infected mice *in vivo*

**Figure 6:**
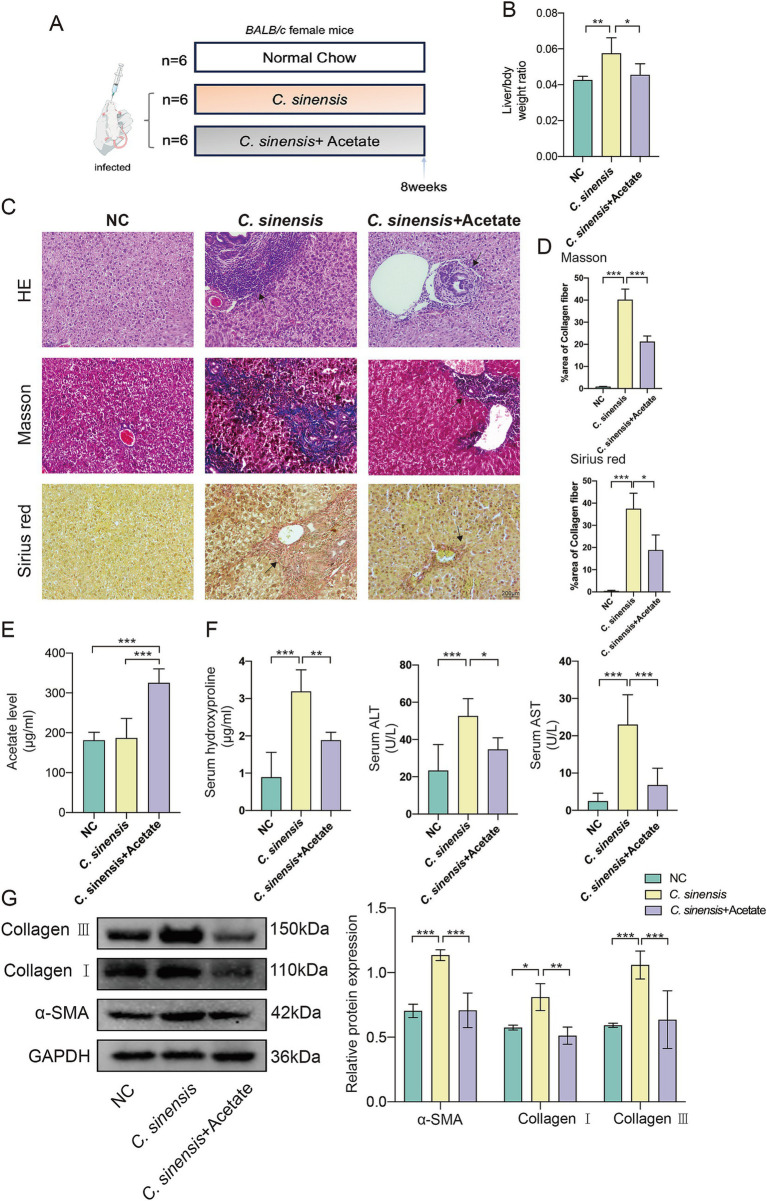
Acetate inhibits the progression of liver fibrosis in *C. sinensis* infected mice. **(A)** Experimental design for acetate treatment in mouse model. **(B)** Liver/body weight ratio in the *C. sinensis* mouse model under different treatment. **(C,D)** The liver pathological sections of three groups of mice were stained (HE, Masson, and Sirius red, scale bar 200 μm), black arrows indicate collagen fiber deposition. And the data for Masson and Sirius red staining are presented as the mean ± SD of three independent experiments. **(E)** Acetate content in mouse serum was measured by ELISA. **(F)** Serum HYP, ALT, and AST levels in mice. **(G)** Expression levels of α-SMA, Collagen I, and Collagen III. Significant differences were analyzed using Ordinary one-way ANOVA **(B,D,E,F)** and two-way ANOVA **(G)**. *p < 0.05, **p < 0.01, ***p < 0.001.

We raised the infected mice for 8 weeks by adding sodium acetate to their daily drinking water (Figure 6A). The results concurred with prior observations, acetate treatment reduced the liver to body weight ratio in mice (Figure 6B), and revealing a marked decrease in liver inflammation in the acetate treated group compared to the *C. sinensis* only group. Masson and Sirius red staining results showed that the collagen deposition around the intrahepatic bile ducts in the acetate group was significantly reduced (Figures 6C,D). Furthermore, the serum acetate levels in the acetate group were notably elevated (Figure 6E), while the serum biochemical markers indicative of fibrosis, namely HYP, ALT, and AST, exhibited a decrease (Figure 6F). Consistent with transcript levels (Supplementary Figure S1B), acetate reduced α-SMA, Collagen I, and Collagen III expression (Figure 6G). These compelling findings underscored the potential of acetate, a functional metabolite of *R. bromii*, to serve as a promising therapeutic agent against liver fibrosis.

### 3.7 Acetate improves gut barrier function

**Figure 7:**
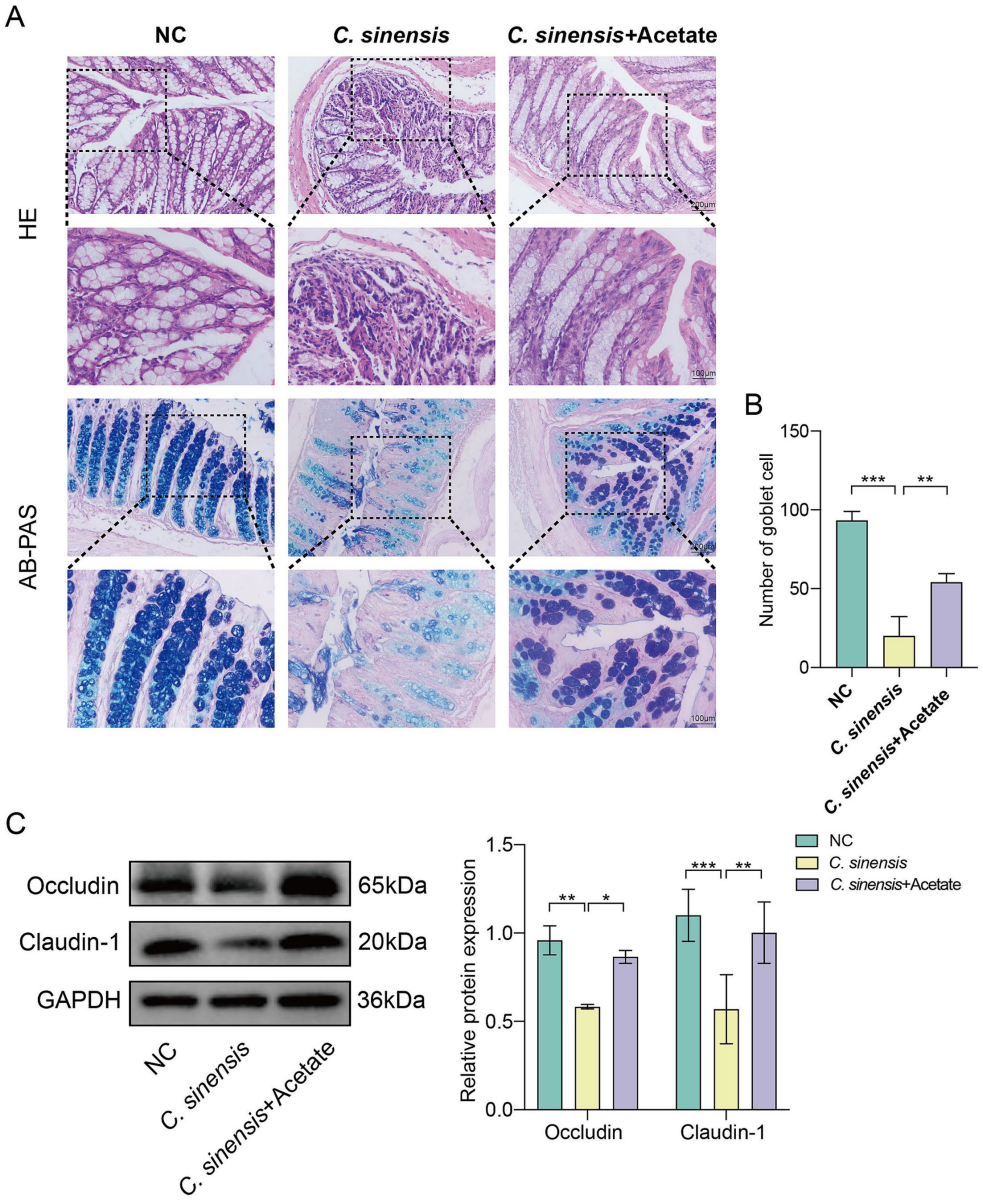
Protective effects of acetate on the intestine. **(A)** Staining of HE and AB-PAS pathological sections of the mouse colon (Scale bar, 200 μm and 100 μm). **(B)** Number of goblet cells in the colon of the three groups of mice. **(C)** Expression levels of intestinal tight junction proteins Occludin and Claudin-1. Significant differences were analyzed using Ordinary one-way ANOVA **(B)** and two-way ANOVA **(C)**. *p < 0.05, **p < 0.01, ***p < 0.001.

Consistent with the previous results, acetate similarly reduced the intestinal inflammatory cell infiltration caused by *C. sinensis* infection, restored the intestinal structure, and increased the number of goblet cells (Figures 7A,B). Compared with *C. sinensis*-infected group, acetate up-regulated the expression of the intestinal tight junction proteins markers Occludin and Claudin-1, and decreased intestinal permeability (Figure 7C). The above results indicated that acetate produced by *R. bromii* restored intestinal barrier function.

### 3.8 Acetate upregulates the PI3K/AKT signaling pathway

**Figure 8:**
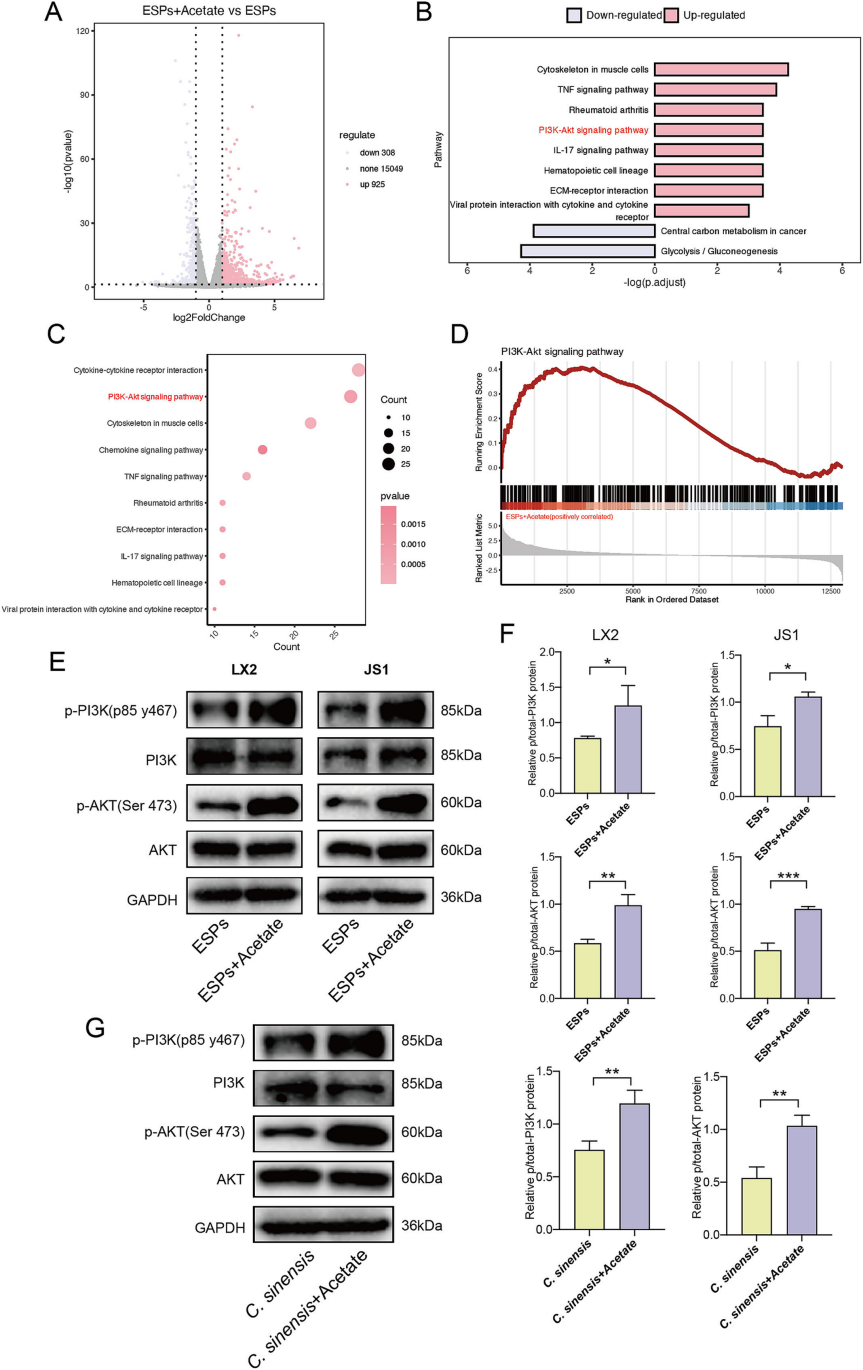
Acetate treatment upregulates the PI3K/AKT signaling pathway. **(A)** Volcano plot of differential genes in ESPs-activated JS1 cell lines with or without acetate treatment (log2 fold change > 1, *p* value < 0.05). **(B,C)** Histogram and bubble plot analysis of differentially expressed genes. **(D)** GSEA of PI3K-AKT signaling pathway in HSC-JS1 with and without acetate intervention. **(E,F)** Expression level and quantitative analysis of PI3K/AKT pathway proteins in two hepatic stellate cell lines. **(G)** Expression level and quantitative analysis of PI3K/AKT pathway proteins in mouse liver tissue. Significant differences were analyzed using unpaired *t* test. *p < 0.05, **p < 0.01, ***p < 0.001.

To reveal the underlying mechanism, RNA sequencing was performed on HSC (JS1) with and without acetate treatment. Our analysis identified 308 differentially down-regulated genes and 925 up-regulated genes were found in the acetate-treated cells compared with the ESPs treatment group alone (Figure 8A). Further analysis revealed that the PI3K/AKT signaling pathway was significantly upregulated after acetate treatment (Figures 8B,C). Gene set enrichment analysis (GSEA) confirmed the upregulation of the PI3K/AKT signaling pathway in acetate-treated HSCs (Figure 8D). We then validated this pathway in two cell models and in mouse animal models. Consistent with the sequencing results, acetate significantly upregulated phosphorylated PI3K and phosphorylated AKT proteins in both HSCs (Figures 8E,F), and acetate promoted PI3K and AKT phosphorylation in animal tissues (Figure 8G). These findings suggested that the protective effect of acetate against *C. sinensis* induced liver fibrosis was mediated at least in through the activation of the PI3K/AKT signaling pathway.

## 4 Discussion

Liver fluke disease is a chronic infectious hepatobiliary disease caused by liver flukes. In the acute phase, the liver undergoes an inflammatory response, and as the infection time increases, the inflammation gradually subsides and fibrosis gradually forms (Kim et al., 2017). Adults of liver flukes parasitize the liver and bile ducts. On the one hand, the immune response caused by the excretion and secretion products of adults is the main factor in liver and bile duct fibrosis (Maeng et al., 2016). On the other hand, adult worms blocking the hepatic bile ducts can cause obstruction of bile excretion, leading to changes in the host’s gut microbiota (Zheng et al., 2017). Microbial changes affecting the gut-liver axis are associated with the progression of chronic liver disease (Zhang et al., 2023). Research had unveiled a noteworthy variation in the abundance of *Akkermansia* bacteria within the gut, contingent upon distinct post-infection phases of liver fluke infestation (Haonon et al., 2021). This observation underscored a dynamic shift in the gut microbiota’s composition throughout the various stages of liver fluke infection. Nevertheless, the direct correlation between this microbial alteration and the onset of liver fibrosis remains an intriguing hypothesis that awaits further validation.

In the present study, we compared the alterations of the gut microbiota in mice infected with *C. sinensis* during the inflammatory and fibrotic phases. Our results revealed profound disparities in the intestinal flora profile of *C. sinensis* infected mice at different time points, in stark contrast to that of their uninfected counterparts. At the genus level, our primary focused centered on the fluctuations of *Alistipes*, *Lactobacillus* and *Ruminococcus*. Remarkably, the abundance of *Alistipe* increased significantly as infection progressed compared to NC group. Intriguingly, a separate study had implicated *Alistipes* in exacerbating colon inflammation in mice and fostering bacterial translocation to the spleen, thereby compromising intestinal barrier integrity (Hajjar et al., 2023). However, an intriguing trend emerged in the *Lactobacillus* population, which showed a surge exclusively at 4 weeks post-infection, mirroring previous research (Kim et al., 2019; Pakharukova et al., 2023). This phenomenon was postulated to be a countervailing mechanism of the body, as *Lactobacillus* is known to interact with intestinal mucosa, fostering Treg cell differentiation and proliferation while secreting anti-inflammatory cytokines like TGF-β and IL-10 (Guo et al., 2023). Initially, *C. sinensis* infection suppresses Treg cells (Yan et al., 2015), but *Lactobacillus* timely intervention may aid in infection establishment while tempering excessive immune responses, thereby restoring equilibrium. Additionally, the abundance of *Ruminococcus* displayed a biphasic pattern, peaking during the inflammatory phase (4W) and tapering off during the fibrotic phase (16W). Given that *R. bromii* predominates within the *Ruminococcus* genus (Kim et al., 2024), we further confirmed this trend by observing a significant depletion of *R. bromii* during fibrosis. This observation underscored the intricate interplay between gut microbiota dynamics and the progression of *C. sinensis*-induced pathologies.

*R. bromii* is a well-known bacteria that degrades resistant starch and is a key commensal bacteria in maintaining the homeostasis of the intestinal microenvironment (Ze et al., 2012). Previous studies had shown that the abundance of *R. bromii* was negatively correlated with the severity of several diseases, including viral hepatitis, primary biliary cirrhosis, and NASH (Duarte et al., 2018; Lv et al., 2016; Zhao et al., 2021). In the genus *Ruminococcus*, *Ruminococcus faecis* (*R. faecis*) had been reported to modulate liver tissue inflammation and reduced the expression of fibrosis markers in MCD-fed mice (Lee et al., 2020). Furthermore, a recent study had revealed that *Ruminococcus torque* (*R. torque*) improved metabolic dysfunction associated fatty liver disease (MAFLD)/ metabolic dysfunction associated steatohepatitis (MASH). This bacterium not only reduced fibrotic area and inflammatory factor expression but also restored triglyceride and total cholesterol levels to normative ranges in mice, thereby highlighting the immense potential of *Ruminococcus* as a therapeutic target for fibrosis (Zhang et al., 2024).

Given the established benefits of *R. bromii* in mitigating liver-related conditions and the broader implications of *Ruminococcus* species in modulating liver health, it is compelling to delve deeper into the gut-liver axis, a vital conduit that facilitates the intricate interplay between these two organs. The intricate bidirectional interaction between the gut and liver is accomplished through the gut-liver axis, a pivotal connection primarily forged by the portal vein (Tripathi et al., 2019). Accumulating evidence underscored the pivotal role that the gut microbiota plays an important role in liver diseases. Our study had unveiled that oral administration of *R. bromii* not only alleviated liver inflammation and fibrosis, but also improved the barrier function and restored the population of goblet cells within the mice colon. Goblet cells are the “sentinels” in the intestine, which secrete a layer of protective mucus (chiefly composed of mucin) on the surface of the intestine to prevent pathogens from directly invading the intestinal wall tissue and triggering inflammatory responses, and maintain intestinal homeostasis (Birchenough et al., 2017). Once the intestinal barrier is damaged or the intestinal permeability is increased, bacteria and their metabolites such as LPS located in the gut may translocate into the blood circulation and reach the liver to activate HSCs, leading to liver injury (Zhang, 2022).

Hepatic stellate cells play an important role in the progression and reversal of liver fibrosis (Trivedi et al., 2022). Our previous studies had found that ESPs could stimulate the activation of primary HSCs, expressed α-SMA, and over-accumulated ECM proteins, thereby promoting the progression of liver fibrosis (Zhao et al., 2023). In the present study, we observed the inhibitory effect of *R. b* CM on HSCs activation *in vitro*, prompting the hypothesis that a specific component produced by *R. bromii* might plays a key role. Subsequent metabolomics analysis, focusing on short-chain fatty acids (SCFAs) showed that *R. bromii* predominantly metabolized acetate, aligning with previous findings (Laverde Gomez et al., 2019). SCFAs, an important class of metabolites produced by intestinal bacteria through the fermentation of dietary fiber and resistant starch, including acetate, propionate, and butyrate. These metabolites not only serve as energy sources for colon cells but also modulate immune responses and regulate the gut-liver axis, thereby exerting profound effects on intestinal health and systemic metabolism (Canfora et al., 2015; Yang et al., 2020). Recent studies had found beneficial effects of acetate in mitigating inflammation, obesity, and cancer. For example, acetate could significantly attenuate hepatic steatosis and inflammation in NASH mice by activating AMPK, inducing the expression of fatty acid oxidation genes in hepatocytes, and inhibiting macrophage aggregation (Deng et al., 2020). In high-fat diet-induced obesity models, acetate supplementation restored the function of obestatin and glucose-6-phosphate dehydrogenase-glutathione dependent antioxidant system, thereby ameliorated obesity and hepatic lipid metabolism disorders (Olaniyi et al., 2022). Furthermore, acetate engaged G protein-coupled receptor 43 (GPR43) on hepatocytes, inhibiting the IL-6/JAK1/STAT3 pathway, inducing apoptosis of NAFLD/HCC tumor cells, preventing tumor formation, and enhancing intestinal barrier function (Song et al., 2023). Intriguingly, another study had demonstrated that acetate can exert its effects within cells independently of GPR43, the representative receptor of SCFAs, by activating AMPK/PPARγ and inhibiting c-Jun signaling pathway to suppress LX2 activation (Li et al., 2021). Our results showed that acetate upregulated PI3K/AKT signaling pathway. The activation of PI3K/AKT pathway plays an important role in reducing liver fibrosis. For example, PI3K/AKT could activate the downstream mTOR pathway and inhibited autophagy in HSCs (Xiu et al., 2021). Upregulation of PI3K/AKT pathway could also induce apoptosis of activated HSCs, thereby promoting the elimination of these cells by the liver to reduce liver fibrosis (Zhao et al., 2021). In conclusion, we hypothesized that the anti-fibrotic effects of acetate *in vitro* and *in vivo* may be partially mediated through PI3K/AKT pathway activation.

**Figure 9:**
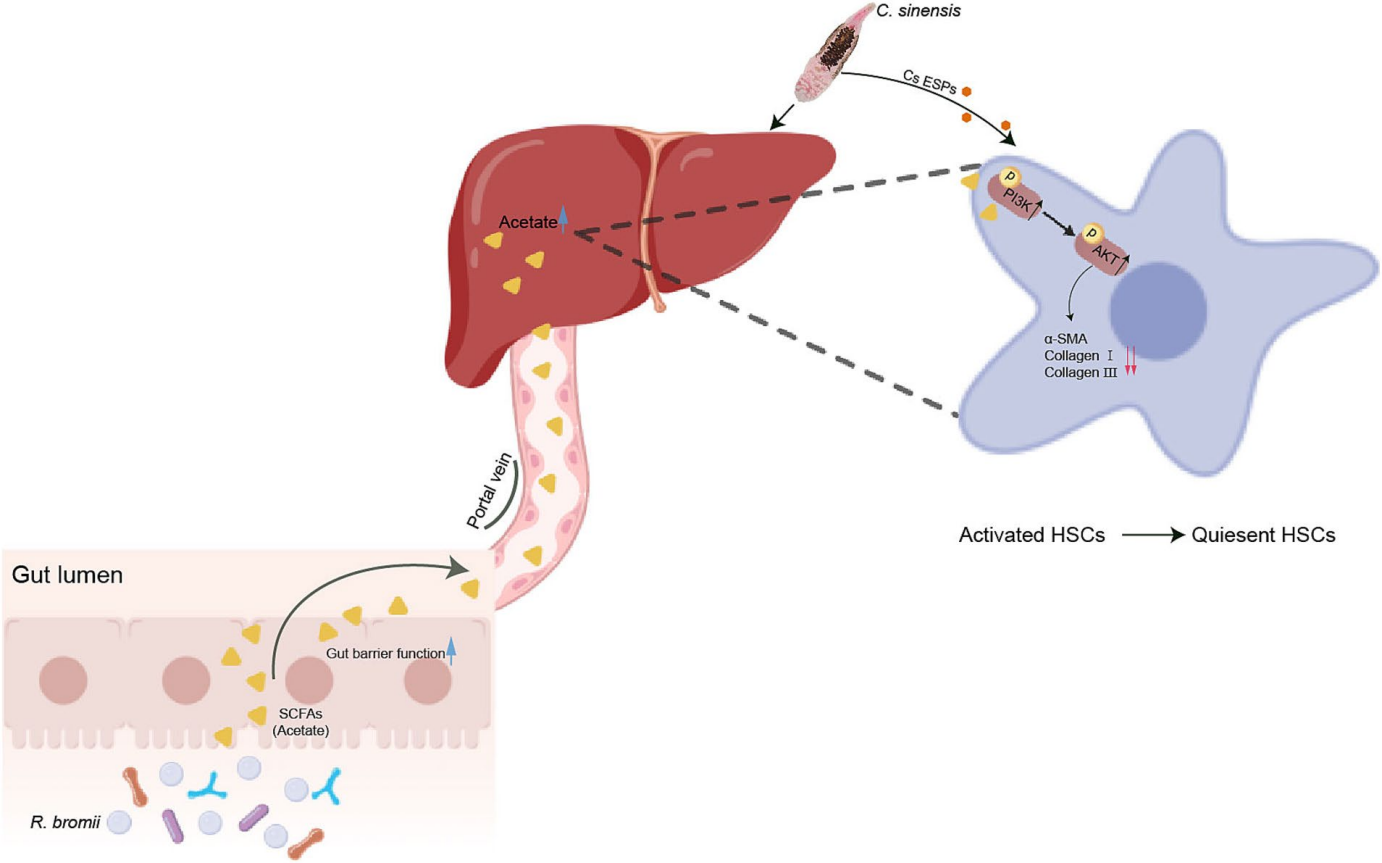
*R. bromii-*generated acetate reaches the liver through the portal vein and upregulates PI3K/AKT signaling pathway to inhibit the activation of hepatic stellate cells, thereby reducing liver fibrosis caused by *C. sinensis*.

Overall, our study demonstrated that *R. bromii* effectively halted the progression of *C. sinensis* induced liver fibrosis by generating acetate via the intricate gut-liver axis, which activating the PI3K/AKT signaling pathway. Furthermore, it inhibited ESPs-induced HSCs activation and ECM accumulation in vitro, demonstrating a profound inhibitory effect. Concurrently, *R. bromii* improved the intestinal barrier function, replenished colonic goblet cell populations, and regulated the inflammatory response, collectively safeguarding against liver fibrosis (Figure 9). This study paves the way for a novel therapeutic avenue in the management of liver fibrosis.

## Data Availability

The datasets presented in this study can be found in online repositories. The names of the repository/repositories and accession number(s) can be found in the article/Supplementary material.

## Supplementary material

Supplementary Figure S1 α-SMA, Collagen I, and Collagen III mRNA relative expression in the livers of mice. **(A)** Different gene transcript levels in the liver tissues of NC, C. sinensis, and R. bromii group. **(B)** Different gene transcript levels in the liver tissues of NC, C. sinensis, and acetate-treated group. Significant differences were analyzed using Ordinary one-way ANOVA. **p* < 0.05, ***p* < 0.01, ****p* < 0.001.

## References

[ref1] Baxter N. T. Schmidt A. W. Venkataraman A. Kim K. S. Waldron C. Schmidt T. M. (2019). Dynamics of human gut microbiota and short-chain fatty acids in response to dietary interventions with three fermentable fibers. MBio 10, e02566–e02518. doi: 10.1128/mBio.02566-18, PMID: 30696735 PMC6355990

[ref2] Birchenough G. M. H. Nyström E. E. L. Johansson M. E. V. Hansson G. C. (2017). A sentinel goblet cell guards the colonic crypt by triggering Nlrp6-dependent Muc2 secretion. Science 352, 1535–1542. doi: 10.1126/science.aaf7419 PMC5148821 27339979

[ref3] Bouvard V. Baan R. Straif K. Grosse Y. Secretan B. Ghissassi F. E.. (2009). A review of human carcinogens—part B: biological agents. Lancet Oncol. 10, 321–322. doi: 10.1016/S1470-2045(09)70096-8, PMID: 19350698

[ref4] Canfora E. E. Jocken J. W. Blaak E. E. (2015). Short-chain fatty acids in control of body weight and insulin sensitivity. Nat. Rev. Endocrinol. 11, 577–591. doi: 10.1038/nrendo.2015.128, PMID: 26260141

[ref5] Choi B. I. Han J. K. Hong S. T. Lee K. H. (2004). Clonorchiasis and cholangiocarcinoma: etiologic relationship and imaging diagnosis. Clin. Microbiol. Rev. 17, 540–552. doi: 10.1128/CMR.17.3.540-552.2004, PMID: 15258092 PMC452546

[ref6] Deng M. Qu F. Chen L. Liu C. Zhang M. Ren F.. (2020). SCFAs alleviated steatosis and inflammation in mice with NASH induced by MCD. J. Endocrinol. 245, 425–437. doi: 10.1530/JOE-20-0018 32302970

[ref7] Duarte S. M. B. Stefano J. T. Miele L. Ponziani F. R. Souza-Basqueira M. Okada L. S. R. R.. (2018). Gut microbiome composition in lean patients with NASH is associated with liver damage independent of caloric intake: a prospective pilot study. Nutr. Metab. Cardiovasc. Dis. 28, 369–384. doi: 10.1016/j.numecd.2017.10.014, PMID: 29482963

[ref8] Guo M. Liu H. Yu Y. Zhu X. Xie H. Wei C.. (2023). *Lactobacillus rhamnosus* GG ameliorates osteoporosis in ovariectomized rats by regulating the Th17/Treg balance and gut microbiota structure. Gut Microbes 15:2190304. doi: 10.1080/19490976.2023.2190304, PMID: 36941563 PMC10038048

[ref9] Hajjar R. Gonzalez E. Fragoso G. Oliero M. Alaoui A. A. Calvé A.. (2023). Gut microbiota influence anastomotic healing in colorectal cancer surgery through modulation of mucosal proinflammatory cytokines. Gut 72, 1143–1154. doi: 10.1136/gutjnl-2022-328389, PMID: 36585238

[ref10] Haonon O. Liu Z. Dangtakot R. Intuyod K. Pinlaor P. Puapairoj A.. (2021). *Opisthorchis viverrini* infection induces metabolic and fecal microbial disturbances in association with liver and kidney pathologies in hamsters. J. Proteome Res. 20, 3940–3951. doi: 10.1021/acs.jproteome.1c00246, PMID: 34270897

[ref11] Hata T. Rehman F. Hori T. Nguyen J. H. (2019). GABA, γ-aminobutyric acid, protects against severe liver injury. J. Surg. Res. 236, 172–183. doi: 10.1016/j.jss.2018.11.047 30694753 PMC6420924

[ref12] Hu F. Hu X. Ma C. Zhao J. Xu J. Yu X. (2009). Molecular characterization of a novel *Clonorchis sinensis* secretory phospholipase a(2) and investigation of its potential contribution to hepatic fibrosis. Mol. Biochem. Parasitol. 167, 127–134. doi: 10.1016/j.molbiopara.2009.05.003, PMID: 19463858

[ref13] Jin Y. Wi H. J. Choi M. H. Hong S. T. Bae Y. M. (2014). Regulation of anti-inflammatory cytokines IL-10 and TGF-β in mouse dendritic cells through treatment with *Clonorchis sinensis* crude antigen. Exp. Mol. Med. 46:e74. doi: 10.1038/emm.2013.144, PMID: 24480801 PMC3909892

[ref14] Keshavarz Azizi Raftar S. Ashrafian F. Yadegar A. Lari A. Moradi H. R. Shahriary A.. (2021). The protective effects of live and pasteurized Akkermansia muciniphila and its extracellular vesicles against HFD/CCl4-induced liver injury. Microbiol. Spectr. 9:e0048421. doi: 10.1128/Spectrum.00484-21 34549998 PMC8557882

[ref15] Kim Y. J. Jung D.-H. Park C.-S. (2024). Important roles of *Ruminococcaceae* in the human intestine for resistant starch utilization. Food Sci. Biotechnol. 33, 2009–2019. doi: 10.1007/s10068-024-01621-0 39130658 PMC11315831

[ref16] Kim J. Y. Kim E. M. Yi M. H. Lee J. Lee S. Hwang Y.. (2019). Chinese liver fluke *Clonorchis sinensis* infection changes the gut microbiome and increases probiotic Lactobacillus in mice. Parasitol. Res. 118, 693–699. doi: 10.1007/s00436-018-6179-x 30623233

[ref17] Kim E. M. Kwak Y. S. Yi M. H. Kim J. Y. Sohn W. M. Yong T. S. (2017). *Clonorchis sinensis* antigens alter hepatic macrophage polarization *in vitro* and *in vivo*. PLoS Negl. Trop. Dis. 11:e0005614. doi: 10.1371/journal.pntd.0005614, PMID: 28542159 PMC5460902

[ref18] Laverde Gomez J. A. Mukhopadhya I. Duncan S. H. Louis P. Shaw S. Collie-Duguid E.. (2019). Formate cross-feeding and cooperative metabolic interactions revealed by transcriptomics in co-cultures of acetogenic and amylolytic human colonic bacteria: Coculture of acetogenic and amylolytic human gut bacteria. Environ. Microbiol. 21, 259–271. doi: 10.1111/1462-2920.14454, PMID: 30362296 PMC6378601

[ref19] Lee Y. A. Wallace M. C. Friedman S. L. (2015). Pathobiology of liver fibrosis: A translational success story. Gut 64, 830–841. doi: 10.1136/gutjnl-2014-306842 25681399 PMC4477794

[ref20] Lee G. You H. J. Bajaj J. S. Joo S. K. Yu J. Park S.. (2020). Distinct signatures of gut microbiome and metabolites associated with significant fibrosis in non-obese NAFLD. Nat. Commun. 11:4982. doi: 10.1038/s41467-020-18754-5, PMID: 33020474 PMC7536225

[ref21] Li W. Deng M. Gong J. Zhang X. Ge S. Zhao L. (2021). Sodium acetate inhibit TGF-β1-induced activation of hepatic stellate cells by restoring AMPK or c-Jun signaling. Front. Nutr. 8:729583. doi: 10.3389/fnut.2021.729583, PMID: 34660662 PMC8515000

[ref22] Lun Z. R. Gasser R. B. Lai D. H. Li A. X. Zhu X. Q. Yu X. B.. (2005). Clonorchiasis: a key foodborne zoonosis in China. Lancet Infect. Dis. 5, 31–41. doi: 10.1016/S1473-3099(04)01252-6, PMID: 15620559

[ref23] Lv L. X. Fang D. Q. Shi D. Chen D. Y. Yan R. Zhu Y. X.. (2016). Alterations and correlations of the gut microbiome, metabolism and immunity in patients with primary biliary cirrhosis. Environ. Microbiol. 18, 2272–2286. doi: 10.1111/1462-2920.13401, PMID: 27243236

[ref24] Maeng S. Lee H. W. Bashir Q. Kim T. I. Hong S. J. Lee T. J.. (2016). Oxidative stress-mediated mouse liver lesions caused by *Clonorchis sinensis* infection. Int J Parasitol 46, 195–204. doi: 10.1016/j.ijpara.2015.11.003 26718397

[ref25] Na B. K. Pak J. H. Hong S. J. (2020). *Clonorchis sinensis* and clonorchiasis. Acta Trop. 203:105309. doi: 10.1016/j.actatropica.2019.105309, PMID: 31862466

[ref26] Olaniyi K. S. Atuma C. L. Sabinari I. W. Mahmud H. Saidi A. O. Fafure A. A.. (2022). Acetate-mediated-obestatin modulation attenuates adipose-hepatic dysmetabolism in high fat diet-induced obese rat model. Endocrine 76, 558–569. doi: 10.1007/s12020-022-03023-w, PMID: 35229234

[ref27] Pakharukova M. Y. Lishai E. A. Zaparina O. Baginskaya N. V. Hong S. J. Sripa B.. (2023). *Opisthorchis viverrini*, *Clonorchis sinensis* and *Opisthorchis felineus* liver flukes affect mammalian host microbiome in a species-specific manner. PLoS Negl. Trop. Dis. 17:e0011111. doi: 10.1371/journal.pntd.0011111, PMID: 36780567 PMC9956601

[ref28] Qi Y. Zheng T. Liu X. Yang S. Li Q. Shao J.. (2023). Sodium acetate regulates milk fat synthesis through the activation of GPR41/GPR43 signaling pathway. Front. Nutr. 10:1098715. doi: 10.3389/fnut.2023.1098715, PMID: 36969813 PMC10035050

[ref29] Song Q. Zhang X. Liu W. Wei H. Liang W. Zhou Y.. (2023). *Bifidobacterium pseudolongum*-generated acetate suppresses non-alcoholic fatty liver disease-associated hepatocellular carcinoma. J. Hepatol. 79, 1352–1365. doi: 10.1016/j.jhep.2023.07.005, PMID: 37459922

[ref30] Tripathi A. Debelius J. Brenner D. A. Karin M. Loomba R. Schnabl B.. (2019). The gut-liver axis and the intersection with the microbiome. Nat. Rev. Gastroenterol. Hepatol. 15, 397–411. doi: 10.1038/s41575-018-0011-z PMC6319369 29748586

[ref31] Trivedi P. Wang S. Friedman S. L. (2022). The power of plasticity—Metabolic regulation of hepatic stellate cells. Cell Metab. 33, 242–257. doi: 10.1016/j.cmet.2020.10.026 PMC7858232 33232666

[ref32] Vital M. Howe A. Bergeron N. Krauss R. M. Jansson J. K. Tiedje J. M. (2018). Metagenomic insights into the degradation of resistant starch by human gut microbiota. Appl. Environ. Microbiol. 84:e01562-18. doi: 10.1128/AEM.01562-18 30266729 PMC6238065

[ref33] Xiu A. Y. Ding Q. Li Z. Zhang C. Q. (2021). Doxazosin attenuates liver fibrosis by inhibiting autophagy in hepatic stellate cells via activation of the PI3K/Akt/mTOR signaling pathway. Drug Des. Devel. Ther. 15, 3643–3659. doi: 10.2147/DDDT.S317701, PMID: 34456560 PMC8387324

[ref34] Xu M. Jiang Z. Huang W. Yin J. Ou S. Jiang Y.. (2018). Altered gut microbiota composition in subjects infected with *Clonorchis sinensis*. Front. Microbiol. 9:2292. doi: 10.3389/fmicb.2018.02292, PMID: 30323795 PMC6172334

[ref35] Yan C. Zhang B. B. Hua H. Li B. Zhang B. Yu Q.. (2015). The dynamics of Treg/Th17 and the imbalance of Treg/Th17 in *Clonorchis sinensis*-infected mice. PLoS One 10:e0143217. doi: 10.1371/journal.pone.0143217 26599407 PMC4658164

[ref36] Yang W. Yu T. Huang X. Bilotta A. J. Xu L. Lu Y.. (2020). Intestinal microbiota-derived short-chain fatty acids regulation of immune cell IL-22 production and gut immunity. Nat. Commun. 11:4457. doi: 10.1038/s41467-020-18262-6, PMID: 32901017 PMC7478978

[ref37] Ze X. Duncan S. H. Louis P. Flint H. J. (2012). *Ruminococcus bromii* is a keystone species for the degradation of resistant starch in the human colon. ISME J. 6, 1535–1543. doi: 10.1038/ismej.2012.4, PMID: 22343308 PMC3400402

[ref38] Zhang P. (2022). Gut microbiota exaggerates triclosan-induced liver injury via gut-liver axis. J. Hazard. Mater. 421:126707. doi: 10.1016/j.jhazmat.2021.126707 34315018

[ref39] Zhang Y. Wang X. Lin J. Liu J. Wang K. Nie Q.. (2024). A microbial metabolite inhibits the HIF-2a-ceramide pathway to mediate the beneficial effects of time-restricted feeding on MASH. Cell Metab. 36, 1823–1838.e6. doi: 10.1016/j.cmet.2024.07.004 39079531

[ref40] Zhang K. Yang J. Chen L. He J. Qu D. Zhang Z.. (2023). Gut microbiota participates in polystyrene microplastics-induced hepatic injuries by modulating the gut–liver Axis. ACS Nano 17, 15125–15145. doi: 10.1021/acsnano.3c04449, PMID: 37486121

[ref41] Zhao L. Li J. Mo G. Cao D. Li C. Huang G.. (2023). Recombinant protein EBI3 attenuates *Clonorchis sinensis*-induced liver fibrosis by inhibiting hepatic stellate cell activation in mice. Parasit. Vectors 16:246. doi: 10.1186/s13071-023-05863-5, PMID: 37480105 PMC10360228

[ref42] Zhao Y. Liu X. Ding C. Gu Y. Liu W. (2021). Dihydromyricetin reverses Thioacetamide-induced liver fibrosis through inhibiting NF-κB-mediated inflammation and TGF-β1-regulated of PI3K/Akt signaling pathway. Front. Pharmacol. 12:783886. doi: 10.3389/fphar.2021.783886, PMID: 34867416 PMC8634482

[ref43] Zhao Z. Qin S. Wang L. Li L. Liu Y. Deng L.. (2021). Correlation between gut microbiota and liver biochemical indicators in patients with chronic hepatitis B. Sheng Wu Gong Cheng Xue Bao 37, 301–311. doi: 10.13345/j.cjb.200279 33501810

[ref44] Zheng S. Zhu Y. Zhao Z. Wu Z. Okanurak K. Lv Z. (2017). Liver fluke infection and cholangiocarcinoma: a review. Parasitol. Res. 116, 11–19. doi: 10.1007/s00436-016-5276-y 27718017

