# Supplementary figures and images for: Ruminococcus bromii-generated acetate alleviated Clonorchis sinensis-induced liver fibrosis in mice
Source: Front Microbiol. 2025 Mar 17;16:1532599. doi: 10.3389/fmicb.2025.1532599 (PMC11955622; doi:10.3389/fmicb.2025.1532599)

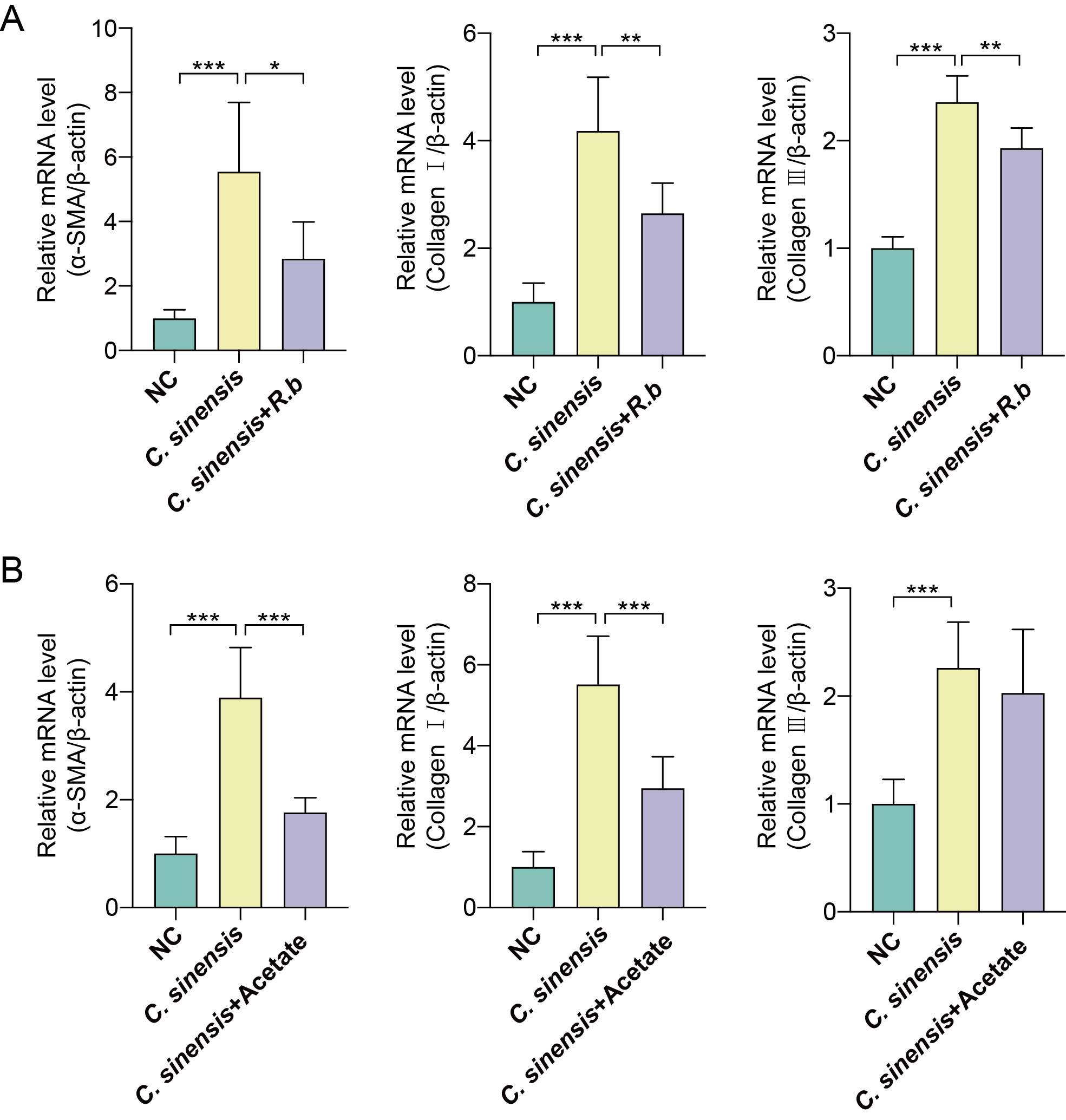

Supplement: Supplementary Figure S1 — α-SMA, Collagen I, and Collagen III mRNA relative expression in the livers of mice. (A) Different gene transcript levels in the liver tissues of NC, C. sinensis, and R. bromii group. (B) Different gene transcript levels in the liver tissues of NC, C. sinensis, and acetate-treated group. Significant differences were analyzed using Ordinary one-way ANOVA. *p < 0.05, **p < 0.01, ***p < 0.001. [file Image_1.jpeg]
